# Couple-Based Intervention to Improve HIV Care Engagement for Women and their Partners in KwaZulu-Natal, South Africa: Outcomes of a Pilot Randomized Controlled Trial

**DOI:** 10.1177/23259582241307694

**Published:** 2025-01-28

**Authors:** Jennifer M. Belus, Alastair van Heerden, Abigail C. Hines, Thembelihle P. Pita, Yvonne Mdakane, Jessica F. Magidson, Heidi van Rooyen, Ruanne V. Barnabas

**Affiliations:** 1 Department of Clinical Research, University Hospital Basel, Basel, Switzerland; 2 University of Basel, Basel, Switzerland; 3 Centre for Community Based Research, Human Sciences Research Council, Pietermaritzburg, South Africa; 4 SAMRC/WITS Developmental Pathways for Health Research Unit, University of the Witwatersrand, Johannesburg, Gauteng, South Africa; 5 Department of Psychology, 1068 University of Maryland, College Park, MD, USA; 6 Center for Substance Use, Addiction, and Health Research (CESAR), 1068 University of Maryland, College Park, MD, USA; 7 The Impact Centre, Human Sciences Research Council, Pretoria, South Africa; 8 Division of Infectious Diseases, 2348 Massachusetts General Hospital, Boston, MA, USA; 9 Department of Medicine, Harvard Medical School, Boston, MA, USA

**Keywords:** couple-based intervention, ART adherence, relationship functioning, pilot study, gender

## Abstract

We evaluated a couple-based intervention targeting human immunodeficiency virus (HIV) care needs of women, with the option to support HIV-related needs of male partners. Adult women with HIV adherence difficulties in a monogamous relationship with a male partner for ≥6 months were recruited in KwaZulu-Natal, South Africa. Twenty couples were randomized (1:1) to either START Together, a five-session manualized behavioral intervention, or treatment as usual, adherence counseling referral. Assessments were completed at baseline, post-treatment, and follow-up. Of the ten couples randomized to START Together, 70% attended at least one intervention session (feasibility); of those, 71% attended all five sessions (acceptability). Independently rated interventionist fidelity was very high (*M* ≥ 2.94 out of 3). Women's self-reported antiretroviral therapy adherence increased similarly in both interventions. For men, self-reported antiretroviral therapy adherence increased up to 25 percentage points in START Together, but not treatment as usual. Findings suggest that START Together may be potentially beneficial for improving HIV outcomes for men.

## Plain Language Summary

*Couple-Based Treatment to Improve HIV Treatment for Women and their Partners in KwaZulu-Natal, South Africa: Results from an Initial Randomized Controlled Study*

We conducted an initial study to test whether a behavioral treatment for couples with the main goal of improving the HIV care needs of women, could also improve the HIV-related needs of male partners. Adult women with HIV who had difficulties with HIV treatment adherence in the past year and who were in a monogamous relationship with a male partner for at least six months were recruited from HIV clinics in KwaZulu-Natal, South Africa. Twenty couples were randomly assigned (1:1) to either START Together, a 5-session intervention based in cognitive-behavioral therapy for couples, or treatment as usual (TAU), a referral to the local HIV clinic for adherence counseling. Assessments were completed at baseline, after the treatment was complete, and at a follow-up timepoint. Of the ten couples that were randomly selected to receive the START Together treatment, 70% attended at least one treatment session (feasibility); of those, 71% attended all 5 sessions (acceptability). Participant self-reported acceptability (Mean =2.93 out of 3) and feasibility (Mean = 2.77 out of 3) were high. An independent person rated the START Together interventionist's delivery of the treatment according to the manual (fidelity), which was determined to be very high (Mean = 2.94 out of 3). Women's self-reported HIV medication adherence increased similarly in both treatment groups. For men, self-reported HIV medication adherence increased up to 25 percentage points in the START Together arm, but not in TAU, with some gains in their intimate relationship as well. Overall, findings suggest that START Together may be potentially beneficial for improving HIV medication adherence and the intimate relationship for men. Future research is needed with a larger sample size and longer-term follow up to confirm these findings.

South Africa currently has 7.6 million people living with human immunodeficiency virus (HIV), of which close to 65% are women.^1^ Its progress toward meeting the Joint United Nations Programme on HIV/AIDS (UNAIDS’) 95-95-95 goals,^2^ which outline the percent of people who are aware of their HIV status, on antiretroviral therapy (ART), and have achieved viral suppression, still requires more efforts to reach the second and third goals. For women with HIV, 80% are on ART and 74% are virally suppressed; for men with HIV, these numbers are slightly lower, with 68% on ART and 62% virally suppressed.^1^ Alternative models of care are likely needed to close these final gaps along the HIV care cascade and meet the UNAIDS goals by 2030.

One approach garnering support in high HIV-burden settings is to work with couples, rather than individuals, to improve HIV treatment outcomes.^3–7^ This approach has proven efficacious in improving HIV testing,^3,4^ preventing vertical transmission of HIV,^5^ and reducing sexual risk behaviors^6,7^ in South Africa specifically, as well as in high- and low-income settings more broadly. Various couple-based interventions that center on HIV prevention have also been adapted and evaluated in the South African context.^7–11^ However, couple-based interventions to improve outcomes of ART initiation, adherence, and viral suppression in the South African context are lacking. In the United States, two studies have examined the effect of a couple-based intervention on ART adherence, with both same-sex and opposite-sex couples, and demonstrated improved adherence.^12,13^ This approach may therefore be useful to test in the South African setting.

Qualitative and quantitative research from South Africa and similar settings in Africa suggest that romantic partners and the relationship context have the potential to play an important, supportive role in ART adherence.^14–18^ For example, partner support was identified as a key factor for ART adherence and care engagement in qualitative studies from South Africa, Tanzania, Malawi, and Zambia.^14,17,18^ This was further supported by quantitative data with Malawian couples, which showed that partner support buffered the negative association of stigma on ART adherence.^16^ At the same time, the dyadic context can also be harmful to HIV care if partners have conflicting or incorrect information about HIV treatment.^19^ Couples who learned about their HIV status jointly in Malawi reported being better able to support each other in subsequent health decisions.^20^ Couple-based interventions targeting ART adherence may therefore be an appealing intervention in this context, increasing social support and a mutual understanding of HIV treatment between partners.

Although there is some local evidence of assortative mating for HIV status in South Africa^21^—that people with HIV are more likely to be partnered with someone who is also living with HIV—romantic partners may still be at different points along the HIV care cascade. Couple-based interventions can therefore also be designed in such a way to address the differing HIV-related needs of both partners within a single intervention, including HIV prevention if one partner is not living with HIV.^22^ If this premise is supported, such interventions may be economically attractive for policymakers.

The goal of the current pilot study was to test the (1) feasibility, acceptability, and fidelity of delivering a couple-based intervention for HIV care, START Together and (2) preliminary efficacy of the intervention on women's ART adherence, men's engagement in HIV care, and the couples’ relationship functioning. We examined the appropriateness of START Together as an exploratory outcome. Given data that suggest men are more likely to engage in healthcare for the benefit of their family (partners or children),^23^ we framed the study as primarily targeting the needs of women, with the option of a flexible intervention to support the HIV-related needs of male partners, should they be interested in focusing on their own HIV-related prevention or treatment needs. We used a pilot randomized controlled trial design to draw firmer conclusions about the intervention's potential efficacy.

## Methods

### Study Design and Sample Size

This study was a pilot parallel (1:1) randomized controlled trial comparing the START Together intervention to treatment as usual (TAU), which included referrals to adherence counseling for women. The study focused on women with HIV and their male partners (regardless of male partner HIV status). We followed the Consolidated Standards of Reporting Trials guidelines for pilot and feasibility trials (see online Appendix 1 for Consolidated Standards of Reporting Trials checklist).^24^ Initially, viral non-suppression was the only ART non-adherence eligibility criterion for women, but this was expanded 3 months after study initiation to include a broader definition of HIV care engagement difficulties (self-report, missed clinic visits, or HIV viral non-suppression), due to difficulties identifying women who were virally non-suppressed in the advent of dolutegravir.^25^ There is also evidence that the presence of any barriers to HIV care is associated with increased mortality in South African women,^26^ which further strengthened the rationale to expand this inclusion criterion.

We enrolled 20 couples (*n* = 10 per treatment arm) as this was the smallest sample size deemed appropriate for any statistical modeling.^27^ Although using a pilot trial to draw conclusions about intervention efficacy is inappropriate,^24^ we planned to examine the direction of effects as a signal of possible efficacy in the future. A detailed description of all study procedures are found in the study protocol paper.^28^

### Setting, Participants, and Procedures

This study took place in the Vulindlela area, a semi-rural area in the province of KwaZulu-Natal. KwaZulu-Natal has the highest HIV prevalence in South Africa,^29^ and the greatest number of people who are food insecure.^30^ Approximately 40% of the province's adult population have completed high school^31^ and approximately one-third of adults are unemployed.^32^

Heterosexual couples (not individuals) were recruited and enrolled in the study. Women were considered the “index participant,” meaning that they were initially identified as potentially eligible and recruited for study participation, followed by the screening of their male partners. Recruitment occurred in clinics providing HIV services. Clinic registries with patients who had missed their monthly clinic appointments to pick up their ART and/or who were virally unsuppressed were reviewed. These potentially eligible women, as well as women who were otherwise attending these participating clinics, were informed about the study, either in-person or telephonically. Interested women were subsequently screened. Male partners of eligible women were then screened telephonically after calling the study phone number.

Eligibility criteria for both members of the couple were as follows: (1) aged 18 or over, (2) currently in a committed, heterosexual, monogamous romantic relationship for at least 6 months, (3) willing to participate in treatment to support the woman's ART adherence, (4) reside in Vulindlela or neighboring community (spending ≥ 4 nights per week in the community), (5) willing to have intervention sessions audio-recorded if randomized to START Together, and (6) able to comfortably communicate in either isiZulu or English. For women participants, additional eligibility criteria were that they needed to be living with HIV, diagnosed at least 3 months prior to study entry, and demonstrate difficulty with past year HIV treatment engagement (self-reported ART adherence difficulties using the Ira Wilson,^33^  ≥ 1 missed clinic visits collected via medical records, or virally unsuppressed (≥ 50 copies/mL based on local standards^34^)).^a^ There were no HIV-related eligibility criteria for men. Couples were excluded for either of the following reasons: (1) report of moderate or severe relationship violence, as measured by endorsing any item on the physical or sexual violence subscales of the World Health Organization Intimate Partner Violence Scale^35^ or (2) ever participated in a couple-based intervention for HIV prevention or treatment.

Couples where both partners screened eligible completed informed consent together, followed by the study baseline assessment. The consent process was conducted with both partners present to ensure that each person understood that they would be participating in the study together as a dyad. The consent process also covered topics specific to couples, such as offering assistance to couples who were experiencing intimate partner violence. Study procedures were also reviewed to make clear which components of the study were completed individually versus as a dyad. Each partner then signed their own consent form. The baseline assessment was subsequently conducted with each partner individually, and each participant was asked again if they were willing to enroll in the study to ensure there was no coercion to participate. Couples were informed that the baseline assessment would determine eligibility for the study. Assessments were conducted at the study field site or local community halls and comprised of self-report measures, medical chart review, and dried blood spot for HIV viral load. Participants were provided with transportation to attend study-related visits.

If after the baseline assessment the couple remained eligible (presence of partner violence was measured during the baseline assessment), the couple was randomized to either TAU or START Together by the project coordinator. The randomization sequence was set up by an independent person not involved in the study and entered into REDCap, the web-based platform used for data entry.^36^ The randomization result was only revealed to staff once “randomize” was selected for a couple's study ID. Couples were subsequently informed about the randomization result over the phone. START Together couples were scheduled as soon as possible for their first session and TAU couples were notified about referrals, with formal referral letters delivered thereafter (see TAU intervention description below).

Subsequent assessments were completed individually (i.e., each partner completed their own assessment independently) at post-treatment and follow-up, which occurred approximately 8-week and 12-week post-randomization, respectively. Assessment windows were, however, flexible to accommodate for difficulty following up with participants, particularly given the pilot nature of the study. Each participant was reimbursed ZAR 120 cash (∼US $8.50) for each completed research assessment. No reimbursement was provided for attending the intervention sessions. Study assessors were not blinded to couples’ treatment condition due to practical constraints of a small research team. The protocol was reviewed and approved by the Human Science Research Council ethics board (REC no. 3/19/09/18).

## Interventions

### START Together

START Together is a 5-session manualized intervention (with the option of three additional booster sessions) based in cognitive behavioral couple therapy applied to HIV treatment,^22^ including problem solving skills for ART adherence following the Life Steps model.^37^ Cognitive behavioral couple therapy is a behavioral therapy approach focused on skills training to improve couples’ communication and problem-solving abilities, with the ultimate goal of improved relationship functioning.^38^ Life Steps is an efficacious single-session problem-solving intervention to improve ART adherence^37^ and has been adapted for use in the South African context^39–41^ and used with couples.^12,42^ Booster sessions were offered to couples who completed all five sessions as a strategy to allow for flexibility in dosage delivered (depending on severity of the presenting problem) and to help determine the preferred number of treatment sessions for future evaluations of the intervention.

We conducted formative work with men and women from the target population and community to understand the local relationship context^43,44^ and the proposed intervention components,^15^ which informed the intervention's development. For example, psychoeducation in the first session of START Together specifically addresses men's role within intervention, which emerged as a potential area of confusion during our formative work. In subsequent sessions of the intervention, couples learn communication and problem-solving skills and actively apply these skills to ART adherence barriers as well as other HIV-related issues. Couples are given practice assignments between sessions to reinforce the skills learned.

The study interventionist was a social worker by training and held a master's degree in research psychology, though this was not a requirement of the position. The interventionist received training in the intervention as well as weekly supervision by the intervention developer (JMB). Intervention sessions were designed to be delivered weekly but in practice depended on the couples’ availability. Sessions were conducted at either local community sites, in a mobile clinic, or at the implementing partner's field site. The interventionist was trained to be aware of the emergence of intimate partner violence (couples were excluded if they endorsed recent violence at baseline) and procedures as well as referrals for this situation were available. A more detailed description of the intervention and supervision can be found in the study's protocol paper.^28^

### TAU

Couples randomized to TAU received referrals to the local clinic for HIV or other healthcare needs. Women in TAU couples were provided with a referral letter specifically for ART adherence support. Men in TAU couples were asked if they needed a referral for HIV services (HIV testing or ART adherence counseling) and were offered referrals for other health services (e.g., mental health, substance use, or other chronic health issues). If interested, one referral letter was provided that included all specific services requested. Participants could take these referral letters to any local healthcare facility where they were receiving care.

## Measures

### Implementation Outcomes

#### Feasibility

We used Proctor’s definition and defined feasibility as the suitability, fit, or utility of the intervention in the current setting.^45^ It was measured using two metrics: (1) the percentage of couples assigned to START Together who agreed to enroll in the intervention (i.e., attend at least one session) and (2) a 14-item feasibility subscale of a validated measure for pragmatic mental health interventions in global low-resource settings,^46^ measured at the post-treatment assessment. Example items on this measure included how difficult it was for participants to leave other duties (e.g., work and parenting) to attend the intervention and whether they had the resources (e.g., phone and airtime) to communicate with their interventionist when needed. The average score across items was used, with higher scores indicating greater feasibility.

#### Acceptability

Following Proctor's model, acceptability was defined as the tolerability or satisfaction of the intervention.^45^ It was measured using three metrics: (1) percentage of couples assigned to START Together who completed all treatment sessions, (2) average number of START Together sessions attended, and (3) a 15-item acceptability subscale of a validated measure for pragmatic mental health interventions in global low-resource settings,^46^ measured at the post-treatment assessment. Example items on this measure included believing that the intervention skills learned were useful, and that the interventionist was able to address questions or concerns about the intervention. The average score across items was used, with higher scores indicating greater acceptability.

#### Fidelity

A pre-defined checklist was developed for each session, ranging between 13 and 17 items, based on the intervention manual. The checklist covered specific session content (ranging from 3 to 7 items) as well as intervention process (e.g., being non-judgmental and engaging both partners throughout the session), which included items relevant to all sessions, such as setting an agenda and assigning homework (10 items). Process items were adapted from a fidelity tool for a cognitive-behavioral couple therapy-based intervention in a high-resource setting^47^ and common therapist factors in low-resource settings.^48^ The interventionist completed a self-report version after each session. In addition, 20% of intervention sessions were randomly selected, translated into English, and rated for fidelity by an independent rater who was trained in the intervention manual and rating scale. Total scores were used, with higher scores on the checklist indicating greater fidelity.

#### Appropriateness

Following Proctor's model, appropriateness was defined as the fit, relevance, or compatibility of the intervention.^45^ This included the intervention's fit with cultural and personal values, appropriate location for intervention delivery, and experiencing the intervention as helpful. It was measured at the post-treatment assessment using the 13-item appropriateness subscale of the assessment tool for pragmatic mental health interventions in global low-resource settings.^46^ The average score across items was used, with higher scores indicating greater appropriateness.

### Preliminary Efficacy Outcomes

#### Women's ART Adherence

This was measured in two ways: (1) viral suppression using the local guideline cutoff <50 copies/mL,^34^ either extracted from medical records (past 30 days) or via dried blood spots and (2) self-reported ART adherence using the normalized average score on the three-item Ira Wilson adherence measure, which ranged from 0 to 100, where higher scores indicate better adherence.^33^ Viral load was collected at baseline and follow-up, and self-reported ART adherence was collected at baseline, post-treatment, and follow-up.

#### Men's Engagement in HIV Care

We assessed the presence of each step of the HIV care cascade: awareness of HIV status (if HIV negative, then needed to receive an HIV test in the past 3 months), prescribed ART, actively engaged in care (attending all scheduled HIV appointments and taking ART as prescribed in the past 3 months), and viral suppression at baseline and follow-up. Each step in the cascade was recorded as either present or absent. All variables except viral suppression were collected via self-report.^b^ We also collected men's self-reported ART adherence using the three-item Ira Wilson (as described under women's ART adherence).

#### Couples’ Relationship Functioning

This construct describes how individuals perceive their romantic relationship and was assessed using the 21-item self-report South Africa Relationship Functioning Assessment (SARFA). The SARFA assesses theoretically relevant relationship factors associated with the intervention, including active relationship building, open communication, and couple-level problem solving.^44^ The average score across items was used, with higher scores indicating more intimate, supportive, and collaborative relationships.^c^ Participants completed this measure at baseline, post-treatment, and follow-up.

## Randomization

The randomization sequence was generated by an independent researcher not directly involved in the study using the randomization module on REDCap, the web-based platform used for data entry.^36^ The randomization was allocated 1:1 to either START Together or TAU. Once a couple was deemed eligible by the research assistant, and confirmed by the study coordinator or principal investigator, the study coordinator clicked “randomize” in REDCap to reveal the couple's treatment arm status. The randomization sequence was not accessible to any team member until this point.

## Data Analysis

We used descriptive statistics to analyze the primary implementation outcomes. We calculated the proportion of the sample meeting the definitions used to define feasibility and acceptability (see Measures section for definitions). We also calculated means, standard deviations, and ranges for the self-report measures of feasibility and acceptability, as well as for fidelity (independent- and interventionist rated).

To evaluate the efficacy outcomes, we used intent-to-treat analyses with all individuals analyzed according to the treatment arm to which they were randomized. A linear mixed model was used to compare the continuous scores for women and men's ART adherence scores and relationship functioning. Men's engagement in the HIV care cascade was examined descriptively. We did not statistically analyze changes in viral suppression as all women (and almost all men) were virally suppressed at both baseline and follow-up. We planned a priori not to include clinical covariates in the analyses, given the small sample size and focus on interpreting the direction of the effects rather than the significance of results. Post hoc analyses conducted to contextualize the results of the planned analyses were as follows: (1) reported level of START Together appropriateness as an additional implementation outcome, (2) referral uptake services by TAU treatment arm, and (3) sub-analysis where the START Together arm only included couples who had attended at least one treatment session to provide information on how at least some exposure to the intervention would impact efficacy outcomes.

## Results

**Figure 1.**
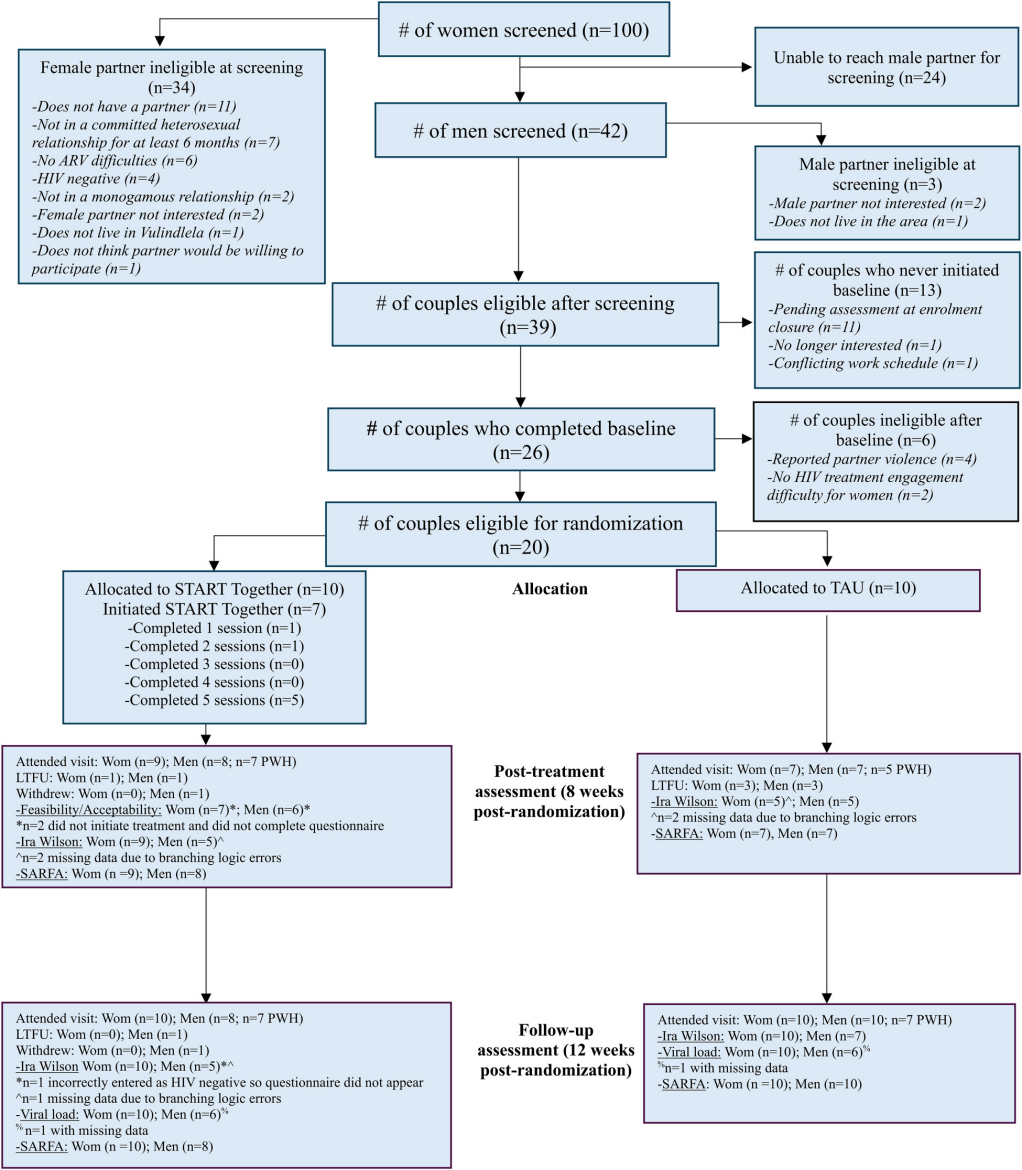
Study CONSORT diagram.

*Note*. Ira Wilson is the measure of ART adherence. LTFU: lost to follow-up; PWH: people with HIV; SARFA: South African Relationship Functioning Assessment.

Figure 1 presents the study flow. Study recruitment began in October 2021 and ended in June 2022. The follow-up assessment data were collected between June and November 2022. In total, 100 women were screened for the study, of which 39 couples were eligible after the screening of both partners. Twenty-six couples completed a baseline assessment, of which 20 couples were eligible and randomized to START Together (*n* = 10 couples) and TAU (*n* = 10 couples). The trial enrollment ended when the proposed sample size was met, and the follow-up assessments ended when all participants had completed their final study assessment or had communicated they would not attend.

The average age of the sample was in their early 40s. The vast majority had less than a high school education and was unemployed, with the majority also reporting food insecurity over the past month. Overall, the demographic and clinical characteristics of the two treatment arms were fairly balanced, as can be seen in Table 1. More prominent differences between the arms include a greater proportion of women in START Together with a high school education or above (50% vs. 10% in TAU) and women in START Together being in their relationship for a shorter amount of time (*M* = 6.6 years vs. 11.1 years in TAU). For men, 90% in START Together were living with HIV and had been diagnosed on average 5.6 years (*SD* = 3.3) prior to study entry. In TAU, 70% of the men reported to be living with HIV and had been diagnosed on average 12.4 years (*SD* = 6.1) prior to study entry.

**Table 1.** Baseline Demographic and Clinical Characteristics of Randomized Couples.

Characteristics	Women		Men	
	TAU	START Together	TAU	START Together
Age, *M* (*SD*)	42.6 (7.8)	41.1 (8.8)	45.8 (13.0)	39.6 (12.3)
Completed high school or above, % (*n*)	10% (1)	50% (5)	40% (4)	20% (2)
Employed, % (*n*)	10% (1)	20% (2)	10% (1)	0% (0)
Relationship length, *M* (*SD*)	11.1 (8.1)	6.6 (3.9)		
Cohabitating, married, or engaged, % (*n*)	70% (7)	50% (5)		
Number of children, *M* (*SD*)	2.8 (1.0)	2.0 (1.3)	3.2 (4.2)	2.2 (1.7)
Number of valuables at home (out of 9), *M* (*SD*)	4.5 (1.6)	5.3 (1.8)	4.5 (1.5)	4.6 (1.6)
Not enough to eat ≥ 1× past month, % (n)	60% (6)	50% (5)	90% (9)	90% (9)
HIV-positive, % (*n*)	100% (10)	100% (10)	70% (7)	90% (9)
Years since HIV diagnosis, *M* (*SD*)	13.8 (6.5)	12.0 (5.5)^a^	12.4 (6.1)	5.6 (3.3)^a^
Partner aware of HIV status	100% (10)	100% (10)	100% (9)	90% (9)

*Note.* TAU: treatment as usual; HIV: human immunodeficiency virus.

^a^ *n* = 1 missing.

### Implementation Outcomes

#### Feasibility

Seven out of 10 couples (70%) randomized to START Together enrolled in the intervention and attended at least one session. The average self-reported feasibility score of the intervention was 2.78 (*SD* = 0.32), with scores ranging from 1.83 to 3.0. Regarding the three couples who did not attend any sessions, one couple ended their relationship before attending any intervention sessions, in one couple the female partner became employed and was unable to attend sessions during the available times offered, and in the third couple, the male partner was no longer comfortable participating in the intervention as he felt he may be stigmatized in the community because the study's implementing organization was known for working with people with HIV. This participant withdrew from the study in its entirety.

#### Acceptability

Of the couples randomized to START Together, 50% (*n* = 5) attended all five treatment sessions. Considering only the couples who initiated the intervention, the percent completing all treatment sessions was 71%. The average number of treatment sessions attended was four, with none of the couples choosing to partake in booster sessions. The average self-reported acceptability score of the intervention was 2.93 (*SD* = 0.21), with scores ranging from 2.21 to 3.0.

#### Appropriateness

The average self-reported appropriateness score of the intervention was 2.87 (*SD* = 0.29), with scores ranging from 1.92 to 3.0.

#### Fidelity

The interventionist conducted 28 sessions as part of the pilot study, and self-rated fidelity was available for all sessions. The interventionist's self-rated fidelity was very high, with an average process fidelity score of 2.99 (*SD* = 0.03, range = 2.89–3.0) and average content fidelity score of 2.97 (*SD* = 0.08, range = 2.67–3.0). The independent rated fidelity from 20% of sessions (*n* = 5) revealed perfect process fidelity scores (*M* = 3.0, *SD* = 0) and a very high content fidelity score of *M* = 2.94 (*SD* = 0.08, range = 2.86–3.0).

#### TAU Referral Uptake

All women in the TAU arm received referrals for ART counseling. Four men in TAU chose a referral when offered: two requested a referral for ART adherence counseling and two requested a referral for support with other chronic health conditions (e.g., diabetes and hypertension). The other six men in TAU declined a referral for any services.

### Preliminary Efficacy Outcomes

#### Women's ART Adherence

All women in the sample at baseline and at follow-up were virally suppressed. Sample means for the Ira Wilson total score at each of the three timepoints is found in Table 2. Women in both treatment arms increased their reported ART adherence between 23 and 25 percentage points at the two follow-up timepoints compared to baseline. For example, the START Together treatment arm reported an average Ira Wilson score of 83.3 (out of 100) at post-treatment and 81.7 at follow-up, compared to 58.5 at baseline. The results of the predictive model for ART adherence, found in Table 3, do not suggest any treatment arm differences. The results are similar for the subsample of START Together women who completed at least one treatment session (see Table 3 and Supplementary Table 1), though the follow-up average adherence was 6 percentage points higher at 87.8 compared to the full sample of START Together women.

**Table 2.** Self-Reported ART Adherence and Relationship Functioning Sample Means and Standard Deviations Over Time by Treatment Arm.

Measure	TAU			START Together		
	Baseline	8 weeks	12 weeks	Baseline	8 weeks	12 weeks
Women Ira Wilson (full sample)	56.7 (19.7)	79.8 (21.0)	80.2 (21.4)	58.5 (30.8)	83.3 (14.8)	81.7 (18.1)
Women Ira Wilson (≥1 START Together session attended)				61.2 (25.2) (*n* = 7)	86.8 (10.9) (*n* = 6)	87.8 (14.5) (*n* = 7)
Men Ira Wilson (full sample)	72.5 (18.7)	66.7 (20.8)	79.7 (13.3)	68.9 (31.8)	91.5 (18.9)	94.0 (13.4)
Men Ira Wilson (≥1 START Together session attended)				64.1 (35.7) (*n* = 6)	89.4 (21.2) (*n* = 4)	94.0 (13.4) (*n* = 5)
Women SARFA (full sample)	5.53 (0.70)	5.68 (0.51)	5.63 (0.46)	5.62 (0.31)	5.12 (1.50)	5.20 (0.84)
Women SARFA (≥1 START Together session attended)				5.75 (0.20) (*n* = 7)	5.68 (0.42) (*n *= 6)	5.49 (0.44) (*n* = 7)
Men SARFA (full sample)	5.22 (0.49)	5.60 (0.18)	4.91 (0.93)	5.23 (0.32)	5.03 (1.35)	4.98 (1.50)
Men SARFA (≥1 START Together session attended)				5.17 (0.30) (*n* = 7)	5.56 (0.16) (*n* = 6)	5.49 (0.29) (*n* = 6)

*Note.* Ira Wilson is a measure of ART adherence. TAU: treatment as usual; SARFA: South African Relationship Functioning Assessment and measures relationship functioning.

**Table 3.** Models Predicting Self-Reported ART Adherence and Relationship Functioning Over Time by Treatment Arm.

Effect	Women Ira Wilson			Men Ira Wilson			Women SARFA			Men SARFA		
	Estimate (SE) [95% CI]	*F* or *t*	*p*	Estimate (SE) [95% CI]	*F* or *t*	*p*	Estimate (SE) [95% CI]	*F* or *t*	*p*	Estimate (SE) [95% CI]	*F* or *t*	*p*
Intercept TAU (baseline)	56.7 (6.9) [42.3, 71.1]	8.27	<.001	72.5 (8.0) [55.3, 89.7]	9.05	<.001	5.5 (0.3) [5.0, 6.1]	21.69	<.001	5.2 (0.3) [4.6, 5.8]	18.22	<.001
ST (intercept)	1.9 (9.7) [−18.5, 22.0]	0.19	.84	−3.6 (10.7) [−26.6, 19.3]	−0.34	.73	(0.4) [−0.7, 0.8]	0.24	.81	0.005 (0.4) [−0.8, 0.9]	0.01	.99
Time *(df)*	2, 30	10.52	<.001	2, 18	5.12	.01	2, 32	0.38	.68	2, 29	1.32	.28
Post-tx for TAU	23.2 (10.2) [2.5, 44.0]	2.28	.02	−5.4 (8.0) [−22.1, 11.4]	−0.67	.50	0.2 (0.3) [−0.4, 0.9]	0.72	.47	0.4 (0.4) [−0.3, 1.1]	1.15	.25
Follow-up for TAU	23.5 (8.0) [7.2, 39.9]	2.94	.006	7.2 (7.1) [−7.7, 22.0]	1.02	.32	0.1 (0.3) [−0.5, 0.7]	0.37	.71	−0.3 (0.3) [−0.9, 0.3]	−1.00	.32
Time×Treatment arm (df)	2, 30	0.00	.99	2, 18	3.41	.05	2, 32	1.68	.20	2, 29	1.08	.35
Post-tx for ST	0.5 (13.1) [−26.3, 27.3]	0.04	.97	27.6 (11.2) [4.0, 51.2]	2.45	.02	−0.7 (0.4) [−1.6, 0.1]	−1.73	.09	−0.6 (0.5) [−1.6, 0.4]	−1.24	.22
Follow-up for ST	−0.4 (11.3) [−23.5, 22.7]	−0.04	.97	19.5 (10.6) [−2.8, 41.8]	1.84	.08	−0.5 (0.4) [−1.3, 0.3]	−1.33	.19	0.1 (0.5) [−0.9, 1.0]	0.14	.88

*Note.* Ira Wilson is a measure of ART adherence. TAU: treatment as usual; ST: START Together; df: degrees of freedom; SARFA: South African Relationship Functioning Assessment and measures relationship functioning; tx: treatment.

#### Men's Engagement in Care

Details of the HIV care cascade for men by treatment arm are found in Table 4. At baseline, 17 men were aware of their HIV status: 16 men were living with HIV and one man was HIV-negative but had tested for HIV in the past 3 months. At follow-up, one HIV-negative man in START Together tested for HIV in the past 3 months and therefore became aware of his status. Of the 16 men living with HIV at baseline, all were prescribed ART, though only 43% in TAU and 55% in START Together were consistently engaged in HIV care. With regard to viral suppression, 88% of the 16 men with HIV were virally suppressed at baseline and 91% at follow-up for the 12 men who had available data, and this was similar between the groups.

**Table 4.** Men's Engagement in HIV Care.

	TAU		START Together	
	Baseline	12 weeks	Baseline	12 weeks
Never tested for HIV	10% (1/10)	10% (1/10)	0% (0/10)	0% (0/10)
HIV-negative, tested for HIV > 3 mos ago	10% (1/10)	10% (1/10)	10% (1/10)	0% (0/10)
HIV-negative, tested for HIV ≤ 3 mos ago	10% (1/10)	10% (1/10)	0% (0/10)	10% (1/10)
Living with HIV	70% (7/10)	70% (7/10)^a^	90% (9/10)	90% (9/10)^a^
Total aware of status	80% (8/10)	80% (8/10)^a^	90% (9/10)	100% (10/10)^a^
Prescribed ART	100% (7/7)	—^b^	100% (9/9)	—^b^
Engaged in consistent HIV care	43% (3/7)	—^c^	56% (5/9)	—^c^
Virally suppressed	86% (6/7)	83% (5/6)^d^	89% (8/9)	100% (6/6)^e^

*Note.* TAU: treatment as usual; ART: antiretroviral therapy; HIV: human immunodeficiency virus.

^a^ Although not all respondents living with HIV returned for the follow-up, those who were positive at baseline were still counted as aware of their status at follow-up.

^b^ Not asked at follow-up for those who reported ART prescription at baseline.

^c^ Substantial data missing due to problem with programming of data collection tool.

^d^ The one unsuppressed viral load at both baseline and follow-up is the same participant.

^e^ The participant with the unsuppressed viral load at baseline was missing data at the follow-up visit.

In terms of self-reported ART adherence (see Table 2), men in the START Together arm reported an average adherence increase ranging from 23 to 25 percentage points, up to an average score of 94.0 (out of 100) at follow-up, compared to 68.9 at baseline. Men in TAU on the other hand showed increases of up to only 5 percentage points, starting from a similar baseline of 72.5. The predictive analyses in Table 3 suggest there may be differences between the treatment arms at the post-treatment assessment (Time×Treatment arm, *p* = .05). The results were similar for the START Together subsample who completed at least one treatment session (see Supplementary Table 1), as compared to the full sample of START Together men.

#### Relationship Functioning

Sample means of women and men's scores on the SARFA by treatment arm are presented in Table 2. The average relationship functioning of both women and men in START Together decreased slightly at the post-treatment and follow-up assessments compared to baseline, by 7%–9% for women and 4%–5% for men. However, when only the subsample who attended at least one treatment session of the START Together intervention were analyzed, women's scores decreased by 1%–5% and men's scores increased by 6%–8% at the follow-ups. Women in TAU's SARFA scores increased by 2%–3% at the follow-up assessments compared to baseline, whereas TAU men had a 7% increase at post-treatment and then a 6% drop below their baseline score at follow-up. Results of the predictive models are presented in Table 3 for the intent-to-treat sample and Supplementary Table 1 for the subsample who attended at least one START Together session. These results suggest that men in START Together who attended at least one treatment session may have seen benefit in their relationship, as compared to men in TAU.

### Harms

There were no serious adverse events reported during the study. One couple randomized to START Together ended their relationship prior to attending any treatment sessions, but reported that their relationship dissolution was not associated with the study. One male participant randomized to START Together withdrew from the study as he was concerned about possible community stigmatization if he became associated with the study's implementing partner organization, which was known for working with people with HIV.

## Discussion

This pilot study provides initial data on implementation and efficacy outcomes of a couple-based intervention targeting women's HIV care engagement alongside the possibility of ancillary benefits for male partners in heterosexual, monogamous couples living in a semi-rural region of KwaZulu-Natal, South Africa. Overall, the data suggest relatively high levels of acceptability of START Together, though there were some barriers to engagement in the intervention. At the same time, the intervention was delivered with high fidelity in both the process and content domains, which is encouraging as working with dyads or families is typically more clinically complex than working with individuals. The preliminary efficacy data suggest that men who participate in START Together may receive benefits in the realms of ART adherence and relationship functioning, whereas these potential benefits were not observed for women.

Seventy-one percent of the couples who began the START Together intervention attended all treatment sessions. Both men and women provided near perfect scores on all self-reported acceptability items, suggesting that couples who began the intervention saw value in it. However, self-reported feasibility was lower than acceptability. Feasibility items that received a lower score were related to session transportation, managing competing demands (e.g., childcare), and maintaining an income. The lower feasibility was underscored by three couples who did not begin the intervention, though the reasons provided were not directly related to START Together itself. Furthermore, none of the couples who completed the intervention decided to continue with booster sessions. The reason for including optional booster sessions was to collect data on preferred treatment length, based on couples’ needs. Although we do not directly know why couples declined to attend the booster sessions, several couples expressed a sense of closure for their problems after completing the core sessions, suggesting that five sessions of the intervention may be adequate. Future iterations of START Together can directly test the appropriate intervention duration and the possibility of booster sessions in the study design, as studies in high-income settings are now doing.^49^

Our findings are similar to the pilot testing of a family-based intervention with lay providers in Kenya in an open trial, where of the 15 enrolled families, five families received either very limited or no treatment at all.^50^ However, a large clinical trial with 334 couples conducted in KwaZulu-Natal showed much higher feasibility—only 8% of the couples randomized to the four-session intervention to increase couples HIV testing did not attend any of the intervention sessions.^3^ However, couples were only randomized after first attending an initial group session. Decisions related to when randomization occurs ultimately impact the chosen study population (e.g., preselecting more motivated participants) and is an important consideration in the potential uptake of such interventions in real-world clinical settings.

In terms of the intervention's potential efficacy, women did not appear to benefit from START Together in either the domain of ART adherence or relationship functioning, in comparison to their TAU counterparts. However, a consistent signal emerged that men who were randomized to the START Together intervention reported an almost 18-percentage point higher ART adherence score at the follow-up assessment compared to men in the TAU arm. In addition, the relationship functioning of men who received at least one session of START Together also increased at the follow-up assessment compared to baseline, whereas TAU men's scores decreased. Other couple-based intervention studies have also found that men benefit more than women,^7,51^ and this may be especially true for men who have less support from their peers.^52^

The lack of a signal demonstrating potential intervention benefit for women may be due to the omission of gender-specific content. A systematic review of interventions to improve women's ART adherence showed that over 80% of published studies did not directly discuss or integrate the concept of gender empowerment or relevant gender-specific concepts into the interventions.^53^ Further, the majority of these interventions had only a modest effect on women's adherence, suggesting the possibility that gendered content could improve treatment outcomes. Although START Together did not explicitly incorporate gender dynamics into the intervention, the impact of local gender norms and expectations on the teaching and learning of intervention skills were discussed during interventionist training. A future version of START Together should consider adding a gender transformative lens, as this has shown some promise in improving other outcomes for women in Africa.^54–56^

Our study findings build upon research demonstrating that dyadic programs, such as couple-based HIV testing and counseling, can increase linkage to care for newly diagnosed members of a couple, which is often the male partner.^57^ Our findings extend this line of research by showing that a couple-based intervention that addresses outcomes later in the HIV cascade can also benefit men. It is important to note that the majority of men in TAU did not take up a referral for HIV or other services when offered, which further highlights the importance of providing an intervention to men rather than relying on use of referrals. A couple-based approach, and its possible benefits for men, may be one strategy to improve other health outcomes, such as reducing intimate partner violence,^58^ which is of significant concern in South Africa.^59^

Data from this trial provide important insights regarding the feasibility of working with couples. We were able to successfully enroll the target sample size of 20 couples, although it took our study team 9 months to meet this goal, suggesting difficulty reaching the target population in this area of KwaZulu-Natal. Most ineligible women did not meet the relationship criteria; of those who were eligible, our team was unable to get into contact with about one-third of the male partners. In addition, several couples who screened eligible were unable to commit to attending the intervention during offered times due to work commitments. This suggests a certain difficulty in the likelihood of reaching and engaging the target population in real-world clinical settings. Future research could test START Together or a similar intervention within an implementation science framework, integrating the intervention as part of clinical services to better understand the patient and partner-level uptake of this approach. For example, this could include offering the intervention at alternative times to meet the needs of couples with work commitments or allowing male partners to join the intervention for only some sessions, which may increase intervention initiation. Such future research should thoroughly capture a wide range of adverse events, including stigma and discrimination, related to participating in a couple-based intervention for HIV.

Study results should be viewed in the context of its strengths and limitations. Study strengths include the use of a randomized design, testing of a novel intervention that was both theoretically and culturally informed, and data collected from both partners on implementation and preliminary efficacy outcomes. However, there are important limitations of the current study, which include logic errors in the data collection tool and participant medical record review across too many clinics, which resulted in missing data in the HIV-related outcomes and reliance on self-report measures. Finally, our study design did not include qualitative interviews due to time and funding constraints. These data would have offered more in-depth information regarding intervention acceptability and feasibility, which should be incorporated into future research.

Overall, this study provides preliminary implementation and efficacy data on the five-session START Together intervention, a couple-based HIV treatment program for women living with HIV and their male partners in KwaZulu-Natal, South Africa. The study findings suggest that the intervention can be delivered with high fidelity by a non-specialist provider. There were some barriers for couples to initially engage in the intervention, but the intervention was deemed acceptable by couples who were able to attend. START Together showed a signal of positive benefits for men's ART adherence and relationship functioning, but not for women. Future research should build upon the START Together intervention to improve men's care engagement throughout the HIV cascade as well as explore ways to enhance the content to improve health outcomes for women.

## Supplemental Material

sj-doc-1-jia-10.1177_23259582241307694 - Supplemental material for Couple-Based Intervention to Improve HIV Care Engagement for Women and their Partners in KwaZulu-Natal, South Africa: Outcomes of a Pilot Randomized Controlled Trial Supplemental material, sj-doc-1-jia-10.1177_23259582241307694 for Couple-Based Intervention to Improve HIV Care Engagement for Women and their Partners in KwaZulu-Natal, South Africa: Outcomes of a Pilot Randomized Controlled Trial by Jennifer M. Belus, Alastair van Heerden, Abigail C. Hines, Thembelihle P. Pita, Yvonne Mdakane, Jessica F. Magidson, Heidi van Rooyen and Ruanne V. Barnabas in Journal of the International Association of Providers of AIDS Care (JIAPAC)

sj-docx-2-jia-10.1177_23259582241307694 - Supplemental material for Couple-Based Intervention to Improve HIV Care Engagement for Women and their Partners in KwaZulu-Natal, South Africa: Outcomes of a Pilot Randomized Controlled Trial Supplemental material, sj-docx-2-jia-10.1177_23259582241307694 for Couple-Based Intervention to Improve HIV Care Engagement for Women and their Partners in KwaZulu-Natal, South Africa: Outcomes of a Pilot Randomized Controlled Trial by Jennifer M. Belus, Alastair van Heerden, Abigail C. Hines, Thembelihle P. Pita, Yvonne Mdakane, Jessica F. Magidson, Heidi van Rooyen and Ruanne V. Barnabas in Journal of the International Association of Providers of AIDS Care (JIAPAC)

## Notes

a. Self-reported difficulties were measured by any of the following: missed ≥ 3 ART doses in the past 30 days; responded between “very poor” to “fair” regarding how good a job they did taking their ART; responded between “never” and “sometimes” regarding how they often they took their ART properly; scored ≤80% on self-rated overall ART adherence.

b. We originally planned to use medical chart data as our primary approach to constructing the cascades. However, there was too much missing data in the chart extraction records to reliably construct the cascades. Self-report data was pre-specified as a backup approach in our protocol.

c. We originally planned to examine subscales of the SARFA but based on empirical evidence [44], the measure is best conceptualized as one-factor of relationship functioning. We therefore did not examine subscales.
