# Supplementary material for: Couple-Based Intervention to Improve HIV Care Engagement for Women and their Partners in KwaZulu-Natal, South Africa: Outcomes of a Pilot Randomized Controlled Trial
Source: J Int Assoc Provid AIDS Care. 2025 Jan 28;24:23259582241307694. doi: 10.1177/23259582241307694 (PMC11773527; doi:10.1177/23259582241307694)
Supplement: sj-docx-2-jia-10.1177_23259582241307694 - Supplemental material for Couple-Based Intervention to Improve HIV Care Engagement for Women and their Partners in KwaZulu-Natal, South Africa: Outcomes of a Pilot Randomized Controlled Trial [file sj-docx-2-jia-10.1177_23259582241307694.docx]

Supplementary Table 1. *Predictive models with START Together subsample who attended at least one session of the intervention*

|  | Women Ira Wilson | | |  | Men Ira Wilson | | |  | Women SARFA | | |  | Men SARFA | | |
| --- | --- | --- | --- | --- | --- | --- | --- | --- | --- | --- | --- | --- | --- | --- | --- |
| Effect | Estimate (SE) [95% CI] | *F* or *t* | *p* |  | Estimate (SE) [95% CI] | *F* or *t* | *p* |  | Estimate (SE) [95% CI] | *F* or *t* | *p* |  | Estimate (SE) [95% CI] | *F* or *t* | *p* |
| Intercept TAU (baseline) | 56.7 (6.2)  [43.4, 69.9] | 9.12 | <.001 |  | 72.5 (8.0)  [54.9, 90.1] | 9.08 | <.001 |  | 5.5 (0.2)  [5.2, 5.9] | 35.26 | <.001 |  | 5.2 (0.2)  [4.9, 5.6] | 31.18 | <.001 |
| ST (intercept) | 4.6 (9.7)  [-16.1, 25.2] | 0.47 | .64 |  | -8.5 (11.8)  [-34.4, 17.4] | -0.72 | .48 |  | 0.2 (0.2)  [-0.3, 0.7] | .91 | .37 |  | -0.05 (0.3)  [-0.6, 0.5] | -.18 | .85 |
| Time *(df)* | 2, 24 | 8.69 | .001 |  | 2, 17 | 5.57 | .01 |  | 2, 26 | .48 | .62 |  | 2, 25 | 2.90 | .07 |
| Post-tx for TAU | 23.2 (10.5)  [1.5, 44.8] | 2.21 | .03 |  | -5.4 (8.0)  [-22.3, 11.5] | -0.67 | .51 |  | 0.2 (0.2)  [-0.2, 0.6] | 1.16 | .25 |  | 0.4 (0.2)  [-0.1, 0.9] | 1.63 | .16 |
| Follow-up for TAU | 23.5 (8.5)  [6.0, 41.0] | 2.78 | .01 |  | 7.2 (7.1)  [-7.8, 22.2] | 1.01 | .32 |  | - 1. (0.2)   [-0.2, 0.4] | .59 | .56 |  | -0.3 (0.2)  [-0.6, 0.5] | -1.43 | .16 |
| Time x Treatment arm *(df)* | 2, 24 | 0.03 | .97 |  | 2, 17 | 3.78 | .04 |  | 2, 26 | 1.10 | .34 |  | 2, 25 | 2.10 | .14 |
| Post-tx for ST | 1.9 (14.9)  [-28.8, 32.7] | 0.13 | .89 |  | 30.9 (12.0)  [5.6, 56.3] | 2.57 | .01 |  | -0.3 (0.3)  [-0.9, 0.3] | -1.14 | .26 |  | -0.01 (0.4)  [-0.8, 0.7] | -.02 | .98 |
| Follow-up for ST | 3.0 (13.2)  [-24.3, 30.3] | 0.23 | .82 |  | 21.9 (10.9)  [-1.1, 44.8] | 2.01 | .06 |  | -0.4 (0.3)  [-0.9, 0.2] | -1.36 | .18 |  | 0.6 (0.3)  [-0.1, 1.3] | 1.81 | .08 |

*Note.* TAU = treatment as usual. ST = START Together. df = degrees of freedom. SARFA = South African Relationship Functioning Assessment and measures relationship functioning. Ira Wilson is a measure of ART adherence.
